# Assignment of a Physical Energy Scale for the Dimensionless Interaction Energies within the PRIME20 Peptide Model

**DOI:** 10.1002/cphc.202400592

**Published:** 2024-10-30

**Authors:** Thomas Kunze, Christian Lauer, Christian Dreßler, Daniel Sebastiani

**Affiliations:** ^1^ Martin-Luther University Halle-Wittenberg Faculty of Natural Sciences II Von-Danckelmann-Platz 4 06120 Halle/Saale Germany; ^2^ Ilmenau University of Technology Ilmenau University of Technology Weimarer Straße 32 98684 Ilmenau Germany

**Keywords:** backmapping, coarse-grained, molecular dynamics simulations, monte carlo simulation, peptide interactions, PRIME20

## Abstract

We present a calibration scheme to determine the conversion factors from a coarse‐grained stochastic approximation Monte Carlo approach using the PRIME20 peptide interaction model to atomistic force‐field interaction energies at full explicit aqueous solvation. The conversion from coarse‐grained to atomistic structures was performed according to our previously established inverse coarse‐graining protocol. We provide a physical energy scale for both the backbone hydrogen bonding interactions and the sidechain interactions by correlating the dimensionless energy descriptors of the PRIME20 model with the energies averaged over molecular dynamics simulations. The conversion factor for these interactions turns out to be around 2 kJ/mol for the backbone interactions, and zero for the sidechain interactions. We discuss these surprisingly small values in terms of their molecular interpretation.

## Graphical abstract

A physical energy scale is presented for the dimensionless interaction energies within the PRIME20 coarse‐grained peptide model. Specifically, the conversion of backbone hydrogen bond energies and sidechain interaction energies into all‐atom structures is investigated.

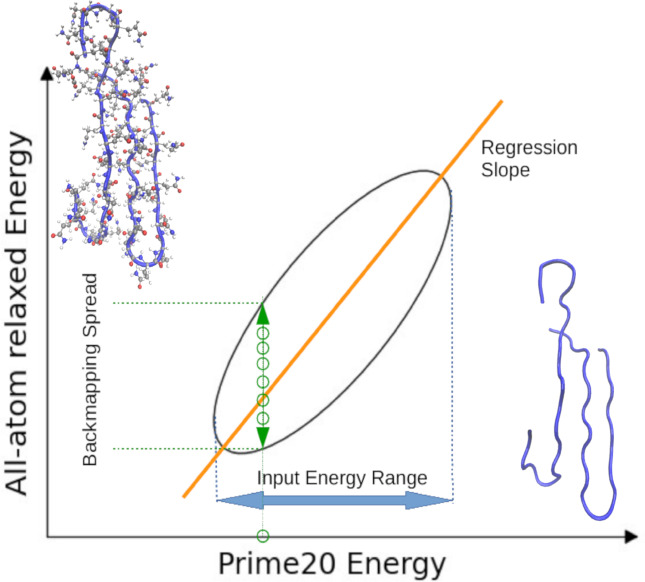

## Introduction

Protein malfunction can lead to various diseases including Alzheimer's,^[1]^ Huntington's,^[2]^ and Parkinson's^[3]^ disease. One problem in this context is the unwanted aggregation of proteins, where the result of that process can lead to the formation of amyloid fibers.[^4^, ^5^]

Computational methods play a crucial role in qualitatively and quantitatively understanding the numerous individual elements of the aggregation process.^[6]^

However, the complexity of aggregation requires the combination of multiple theoretical methods to achieve accuracy while maintaining reasonable timescales.

In our previous work,^[7]^ we provided a protocol that allows the transfer of bio‐molecular systems of intermediate size between two specific simulation methods. This approach combines two different resolution levels (atomistic vs coarse‐grained) and two different interaction potentials (bio‐molecular force fields vs hard‐sphere potentials). Combining these two methods addresses the representability and transferability problems of the quasi‐global coarse‐grained (CG) sampling by local spatio‐temporal phase space coverage of the classical force field molecular dynamics (MD) simulations.[^8^, ^9^, ^10^, ^11^, ^12^, ^13^, ^14^] In detail, our approach combines an MC sampling scheme based on the P20 protein model with MD simulations to regain atomistic accuracy by reintroducing energetic and entropic contributions neglected by the CG potential. Furthermore, explicit solvent interactions may result in a more thermodynamically accurate weighting of the conformations.

Both MC and MD simulations have been extensively used in the past to study biomolecules.[^15^, ^16^, ^17^, ^18^, ^19^, ^20^] As they are highly complementary techniques, several hybrid approaches already combine these two methods.[^21^, ^22^, ^23^, ^24^, ^25^, ^26^, ^27^] Monte Carlo methods are a suitable tool for exploring large parts of the conformational space of biomolecules.

Meanwhile, MD simulations can model the local structural fluctuations and dynamics of a given peptide configuration. By starting from structures obtained from the Monte‐Carlo method, the subsequent MD simulations will provide the atomistic view, further enhanced by explicit water solvation. This allows for the examination of the dynamic characteristics of hydrogen bond networks by automatically including the entropic effects of atomistic degrees of freedom.

## Computational Methods

### Stochastic Approximation Monte‐Carlo Simulation

The Stochastic Approximation Monte‐Carlo (SAMC)[^28^, ^29^] method, which was developed as a mathematical formulation of the Wang‐Landau^[30]^ algorithm, was used for the simulation of a *Glu*_26_‐dimer. The objective of the SAMC is to achieve a flat visitation histogram of energy states. This approach avoids the problem of getting stuck in local energy minima, that can occur with standard MC simulations. The SAMC achieves an even visitation of energy states by approximating the microcanonical density of states (DOS) *g*(*U*) with respect to the potential energy *U*. The DOS describes the number of states in the system that belong to a given energy interval [U,U+ΔU] . SAMC then uses the DOS in its acceptance criterion: for an SAMC move from configuration *x* with energy *U*(*x*) to configuration 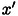 with the energy 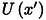 , the move is accepted with a probability of:
(1)

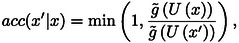




with $\tilde{g}\left(U\right)$ being the current estimate for the DOS. After the move is rejected or accepted, $\tilde{g}\left(U\right)$ is updated according to:

(2) $$\tilde{g}\left(U\left(x_{\mathrm{new}}\right)\right)=\tilde{g}\left(U\left(x_{\mathrm{new}}\right)\right)+\gamma_{t},$$

where 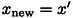 if the move was accepted and $x_{\mathrm{new}}=x$ if the move was rejected. The modification factor *γ_t_* goes to 0 for time t→∞ , according to:

(3) $$\gamma_{t}=\min\left(\gamma_{0},\frac{t_{0}}{t}\right),$$

with *t* being measured in MC steps. The convergence of the SAMC algorithm was proven when additional conditions were fulfilled.[^28^, ^29^, ^31^] Simulations were run until a sufficiently accurate *g*(*U*) was obtained, with $\gamma_{t}<10^{-7}$ . Afterwards, multiple production MC runs with a fixed DOS and over $10^{9}$ MC steps each were performed to collect configuration snapshots over the system's entire energy range.

Four different MC move types were used in the SAMC simulations. Firstly, a local displacement move, which moves a single bead in a randomly chosen direction by a random distance, with a maximal displacement of 0.02 Å. Secondly, a pivot rotation move, which randomly chooses a residue and rotates either its Ψ or Φ angle by a random amount and direction. Additionally, two moves are implemented to manipulate the relative position of the two chains in the system: a whole‐chain rotation and a whole‐chain translation move. After every move, the new configuration must be in agreement with the PRIME20’s constraints on bond lengths and excluded volumes. Similar to already successful calculations,^[32]^ we simulated polyglutamine dimer systems with a chain consisting of 26 glutamine residues. A cubic simulation box with length *L*=150 Å was used, which was periodic in all directions. This translates to a millimolar concentration, which is close to in vitro experiments on polyglutamine aggregation.

In the PRIME20 model, there are peptide backbone‐backbone interactions of amplitude one as well as sidechain‐X interactions (X=backbone or sidechain) of amplitude 0.08. In the concept of this coarse‐grained interaction model, no specific microscopic nature of these interactions is specified, which means both hydrogen bonding and hydrophobic interactions are represented by this effective interaction strength. In our system, however, all three interaction types (peptide backbone‐backbone, sidechain‐backbone or sidechain‐sidechain) are actually hydrogen bonds. The PRIME20 interaction model contains two distinct types of intra‐ and inter‐peptide interactions: backbone hydrogen bonds and sidechain interactions. These interaction types contribute 1.0 and 0.08 arbitrary energy units to the PRIME20 total energy expression, respectively, for each molecular group that actually interacts in the local geometry of a given glutamine structure:

(4) $$E_{P20}=-1N_{\mathrm{backbone}}-0.08N_{\mathrm{sidechains}}.$$

In order to adequately sample this “space of interactions” contained in the ensemble of coarse‐grained structures generated by the MC simulations, we have generated subsets of conformations in such a way that each pair of values for the amplitude of the two interaction types (*N*_backbone_, *N*_sidechains_) is well represented in the ensemble of configurations used as input for our inverse coarse‐graining protocol.

### Molecular Dynamics Simulation

**Figure 1:**
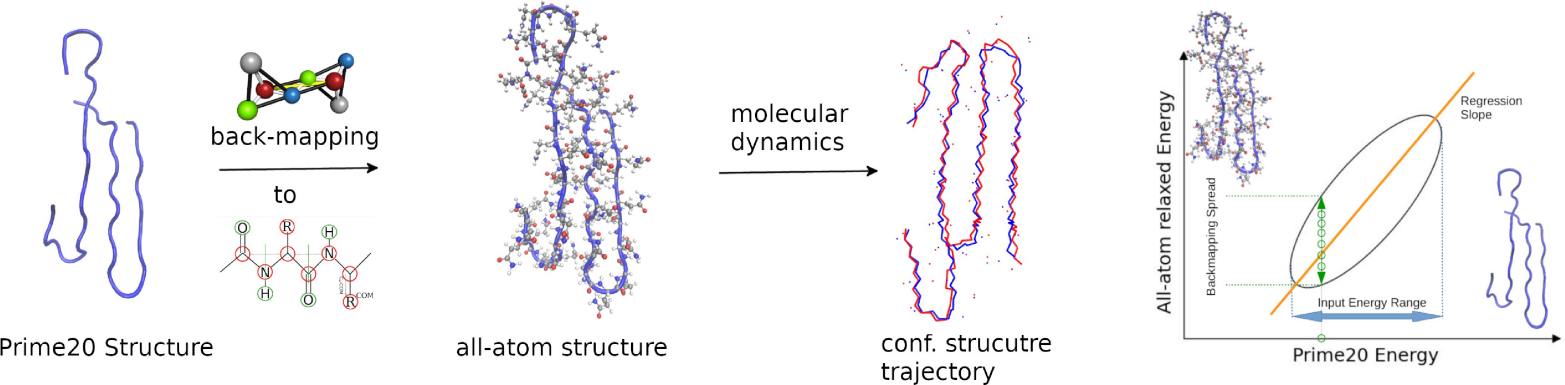
The central process for the generation of data in this article is visualized: starting by back‐mapping^[7]^ Prime20 structures to all‐atom structures and running short MD simulations to compare the energies for both techniques.

In previous work, a protocol for the back‐conversion of conformations obtained from the coarse‐grained peptide interaction model PRIME20 to atomistic structures was developed. The PRIME20 scheme provides simulation data which contains coordinates for the backbone carbon and nitrogen atoms, as well as the center of mass (COM) coordinates of the side chain residues of the peptide, which are indicated by red circles in Figure 1. The atoms labeled with green circles are not provided, however with our previously published algorithm, we derive the coordinates of the carbonyl oxygens and the nitrogen protons in the peptide backbone directly from the backbone carbon coordinates by assuming planar NH‐C‐CO geometry. For the sidechain R, which is only one bead provided in the PRIME20 model, the coordinate of the initial carbon atom is computed by adjacent NH and CO groups, and the orientation of the residue is defined by the connection vector from the backbone C_*α*_ atom to the center of mass from the PRIME20 simulation data. We assume molecular equilibrium conformation for the amino acid residues, so that the anchor point (via the center of mass) and the orientation (via the C_*α*_‐COM vector) are sufficient to reconstruct the coordinates of the full residue.

The atomic coordinates of the *Glu*_26_‐dimer computed this way lead to considerable misalignments in the 3D structure of the peptides. The most common problem is that atoms from two adjacent amino acid residues are too close to each other. However, the protocol turned out to yield reasonable values for the start of a short geometry optimization cycle. The standard optimization algorithms are able to respond to close‐proximity misalignments and reorient the amino acid residues away from each other while maintaining the overall peptide structure proposed by the coarse‐grained scheme. It should be noted that while the resulting atomistic peptide geometry is technically possible, it is not guaranteed that this conformation is locally stable from a thermodynamical perspective. The latter aspect was addressed in our previous work of the back‐mapping scheme.^[7]^

For each PRIME20 energy data point, a *Glu*_26_‐dimer structure was randomly selected from the provided MC structure set and converted into an all‐atom structure, similar to previous research. More specifically, the coarse‐grained structures resulting from the PRIME20 MC simulations were translated into all‐atom structures with both termini charged and were directly suitable for calculations. These structures were then explicitly solvated with 6700 water molecules using the standard GROMACS[^33^, ^34^] solvation tool. After an initial energy minimization (emtol=100; emstep=0.1; niter=20) for all atoms, a 10 ns NVT MD simulation with a 0.5 fs time step was performed at 300 K using velocity rescaling with a 0.1 ps time constant, Lincs 4th order constraint^[35]^ for covalent hydrogen bonds, and the AMBER03^[36]^ force field, while water interactions were represented by the TIP3P^[37]^ water model. The Verlet cutoff scheme and periodic boundary conditions were used, and electrostatics were calculated with PME using potential‐shift Verlet for the Coulomb modifier.

As a reference simulation, 6700 water molecules were simulated with the same MD parameters, but with a slightly smaller box to achieve a similar density. The average energy obtained was −215346 kJ/mol. A short MD simulation of a single ${\mathrm{Glu}}_{26}$ ‐peptide resulted in an average energy of −5392 kJ/mol. Therefore, our simulation with 6700 water molecules and 2 peptides has a reference energy of −226130 kJ/mol. This reference energy was used for visual clarity in our plots.

## Results

### Density of States of the Coarse‐Grained Conformational Space

**Figure 2:**
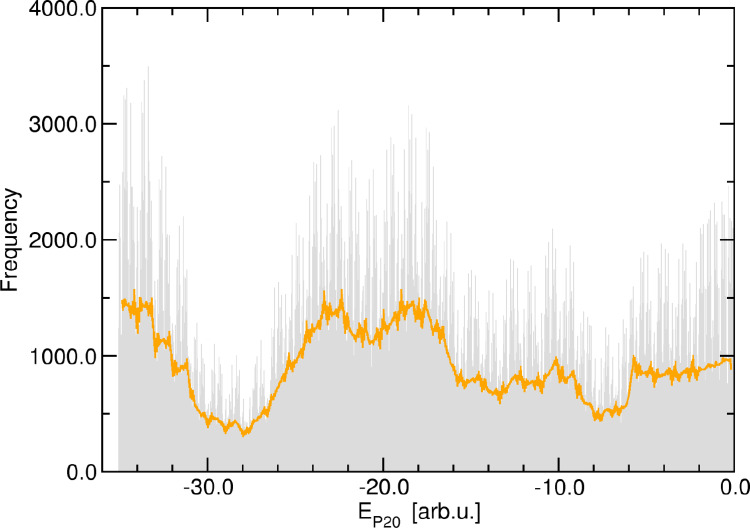
Distribution of the (dimensionless) energies *E*_*P*20_ of the ensemble of coarse‐grained structures (N≈800000 ) generated using the PRIME20 interaction model. The orange line is the running average (for an energy window of 0.5 arb.u.).

We have computed the energy histogram of the ensemble of initial coarse‐grained structures that were generated with the flat‐histogram Monte‐Carlo sampling scheme at the PRIME20 level of theory (see Figure 2). We use the dimensionless energy units provided by the PRIME20 interaction model, which combine inter‐peptide backbone hydrogen bonding and side chain interaction energies with specific relative weights. Although the distribution is not strictly flat, it has no characteristic internal structure, and shows that the sampling protocol provides a sufficient number of conformations for any given energy value. In order to exclude any hidden bias in this distribution, we also analyzed its Fourier transform (see SI for details), which revealed no particular spectral features.

In the PRIME20 model, the total energy (E_P20_) is composed of a larger contribution due to backbone hydrogen bonding and a smaller contribution due to side chain interactions, with a non‐trivial commensurability, see the Methods section. To verify the balanced distribution of the weaker side‐chain interactions contributions, we have additionally calculated the density of states of the total PRIME20 energies E_P20_ *modulo* the hydrogen bonding contributions (i. e. considering only the side chain interactions, represented by the fractional part of E_P20_). This projected density of states is given in the Supporting Information. Again, this distribution function shows no distinct spectral peaks, indicating an adequate statistical representation of all amplitudes for this weaker interaction type.

This preliminary statistical analysis of the underlying conformational space of our peptide dimer in terms of its energy distribution shows that the there are no “forbidden” energy ranges with low densities of states. In particular, also the thermodynamically unfavourable conformations (i. e. those with energies near E_P20_=0 arb.u.) are well represented in the manifold. In this sense, we are confident that our basic data is reasonably unbiased and does not need to be weighted or corrected a posteriori.

Hence, we conclude that the initial Monte‐Carlo sampling at the PRIME20 level can be considered converged for our purposes.

### Energy Correlation between Coarse‐Grained and Atomistic Models

The central goal of this work is to investigate the correlation between the dimensionless energies of the coarse‐grained peptide structures generated under the PRIME20 model and the (regular dimensional) energies of the locally relaxed all‐atom conformations. The all‐atom energies are obtained from our reverse coarse‐graining protocol^[7]^ by means of a preliminary geometry‐optimization and a subsequent 10 ns molecular dynamics simulation (at constant ambient temperature) at the all‐atom force‐field level. The instantaneous total energy values during the MD simulation are then averaged, yielding the final energy value at the all‐atom level. Such a correlation allows to assign an effective physical energy value to the dimensionless energy scale used by the coarse‐grained interaction model.

**Figure 3:**
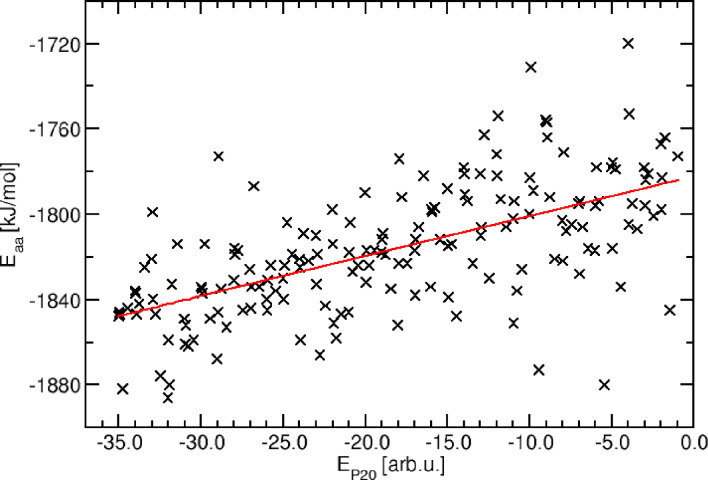
Correlation of *E*_*P*20_ and *E_aa_* for the hydrogen bond series with linear regression analysis. The slope of the regression is 1.9 [kJ mol^−1^/arb_P20_.u.].

The raw correlation as well as the linear fit are shown in Figure 3. Clearly, a positive correlation is recognizable, i. e. structures with more positive PRIME20 energies correspond to conformations with more positive force‐field energies. However, the variations of the final all‐atom energies are quite large, and even exceed the systematic dependence of *E*_aa_ on $E_{P20}$ . It should be noted that there is of course also a statistical error bar associated with every single data point *E*_aa_; this aspect will be addressed later on in this article.

The correlation between coarse‐grained PRIME20 energies $E_{P20}$ and locally relaxed all‐atom conformations *E*_aa_ is obtained as 1.9 kJ/mol per PRIME20 energy unit. At first sight, this value is considerably lower than the typical energy of a hydrogen bond of 20 kJ/mol (one energy unit in the PRIME20 interaction model corresponds to one intermolecular NH⋅⋅⋅OC peptide hydrogen bond). However, the reference situation is not simply a broken peptide hydrogen bond. Instead, both the NH and CO hydrogen bonding partner will establish hydrogen bonds to liquid water from the solvent, but in turn break a water‐water hydrogen bond. The true situation is of course even more involved, as the coordination numbers of the water molecules may differ between the two situation (i. e. a water can donate two hydrogen bonds to other water molecules, but is less likely to bond).

Thus, one PRIME20 energy unit corresponds to the difference between these two competing situations:

(5) $${\Delta E}_{P20}=E_{\mathrm{MD}}\left(\mathrm{Pep}\cdots \mathrm{Pep}\right)+E_{\mathrm{MD}}\left(H_{2}O\cdots H_{2}O\right)\;\;\;-2E_{\mathrm{MD}}\left(\mathrm{Pep}\cdots H_{2}O\right).$$

Hence, a comparably small value of 1.9 kJ/mol makes perfect sense as the effective intermolecular peptide hydrogen bond energy difference.

However, the problem remains that different coarse‐grained structures with virtually no energy difference (e. g. one PRIME20 energy unit) typically yield all‐atom conformations that exhibit considerable energetic deviations (of ten times the corresponding all‐atom energy difference, i. e. 10×2 kJ/mol=20 kJ/mol). This variability represents a challenge for the physical interpretation of the energy landscape generated and sampled by the PRIME20 interaction model; most likely, the coarse‐graining approach suffers from not recognizing many of the more subtle energetic effects of structural deformations of the peptides. Examples of such effects include torsional and angular potentials along the peptide backbone chain, but also steric effects related to the actual size of solvent molecules (e. g. an area with space for 1.9 water molecules can only be filled with one water molecule, which in an all‐atom description will result in a force that tending to reduce the volume of that area).

### Sidechain Interaction

**Figure 4:**
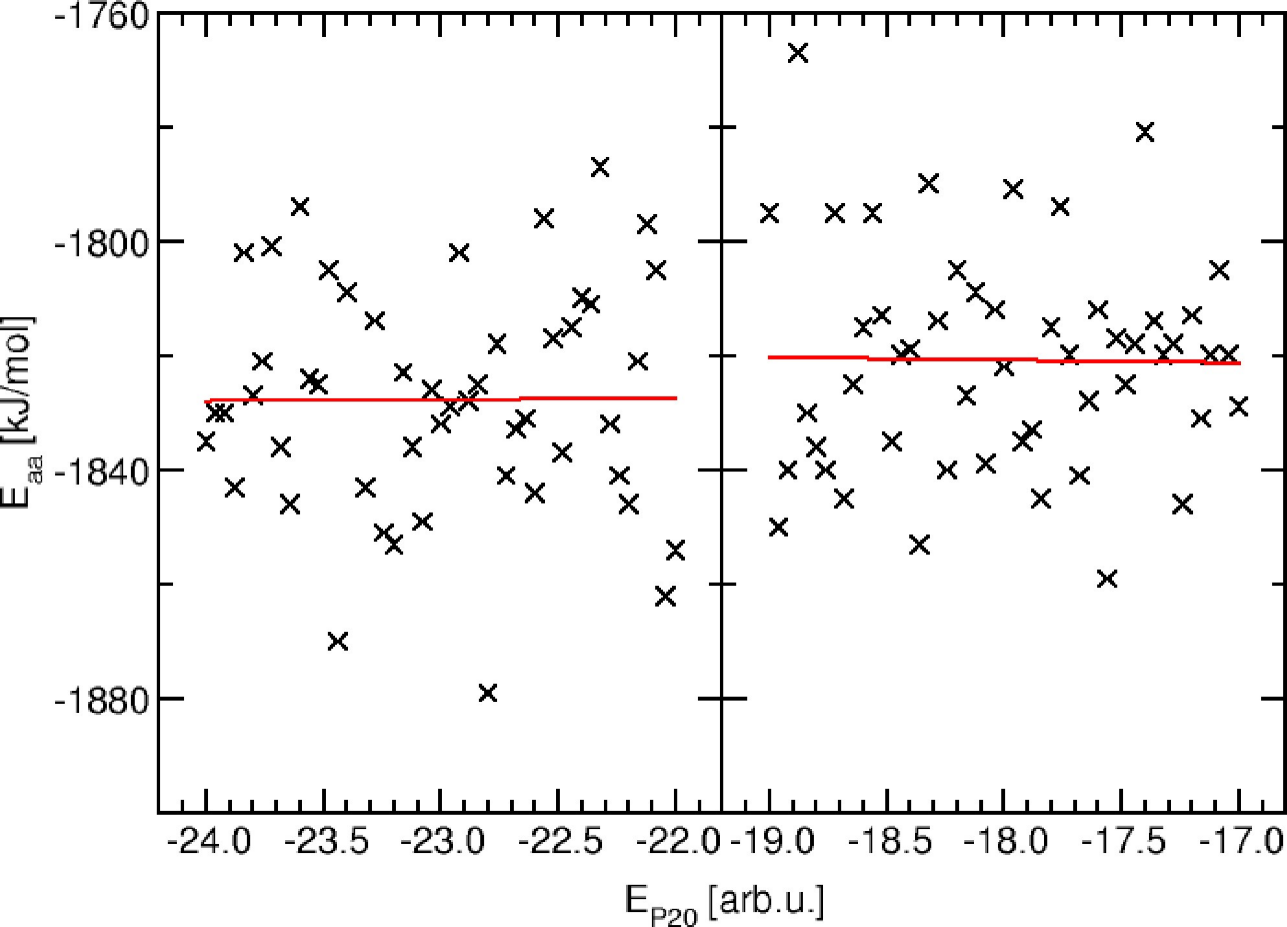
Correlation between the coarse‐grained ($E_{P20}$ ) and atomistic ($E_{\mathrm{aa}}$ ) energies for a series of conformations with identical backbone hydrogen bonding states (at the coarse‐grained level, here corresponding to 22 and 17 hydrogen bonds, respectively, for the left and right plots). In turn, the number of sidechain interactions varies and corresponds to −1/12 units of $E_{P20}$ per sidechain interaction.

In Figure 4, we plot the energies at the coarse‐grained and at the atomistic level for a series of conformations that have an identical number of backbone hydrogen bond interactions, characterized by “large” energy steps (one arbitrary unit) at the coarse‐grained level, but different numbers of sidechain interactions, characterized by “small” energy steps (1/12 of an arbitrary unit). Each of these conformations was processed through our inverse coarse graining protocol, so that each atomistic energy represents an average value obtained during a 10 ns molecular dynamics simulation. Here, we have arbitrarily chosen two specific values for the number of backbone hydrogen bond interactions (22 and 17, respectively, for the two plots in Figure 4).

We observe a correlation between the (coarse‐grained) sidechain interactions and the atomistic energies with practically zero slope. While the atomistic energies are statistically quite scattered with a distribution width of around ±20 kJ/mol, the correlation slope is below 1 kJ/mol per $E_{P20}$ energy unit in both cases. The reason for this weak correlation is that it is statistically challenging to detect a correlation of the order of 1/12 of a hydrogen bond (identified in the previous section as corresponding to an atomistic energy of 2 kJ/mol, resulting in 0.2 kJ/mol for the expected sidechain interaction) in the presence of numerical noise of the order of 20 kJ/mol. From a chemical perspective, even the short MD simulations within our equilibration protocol (10 ns) result in conformational changes that are energetically more important than a single sidechain interaction energy. Hence, we consider the actual energetic conversion factor of the PRIME20 sidechain interactions to be zero. Notably, this does not mean that the sidechain interactions have zero interaction strength, but rather that the correlation of the PRIME20 interaction scheme with the true (atomistic) interaction energy is small.

### Analysis of Statistical Errors/Numerical Uncertainties of the Atomistic MD Simulations

**Figure 5:**
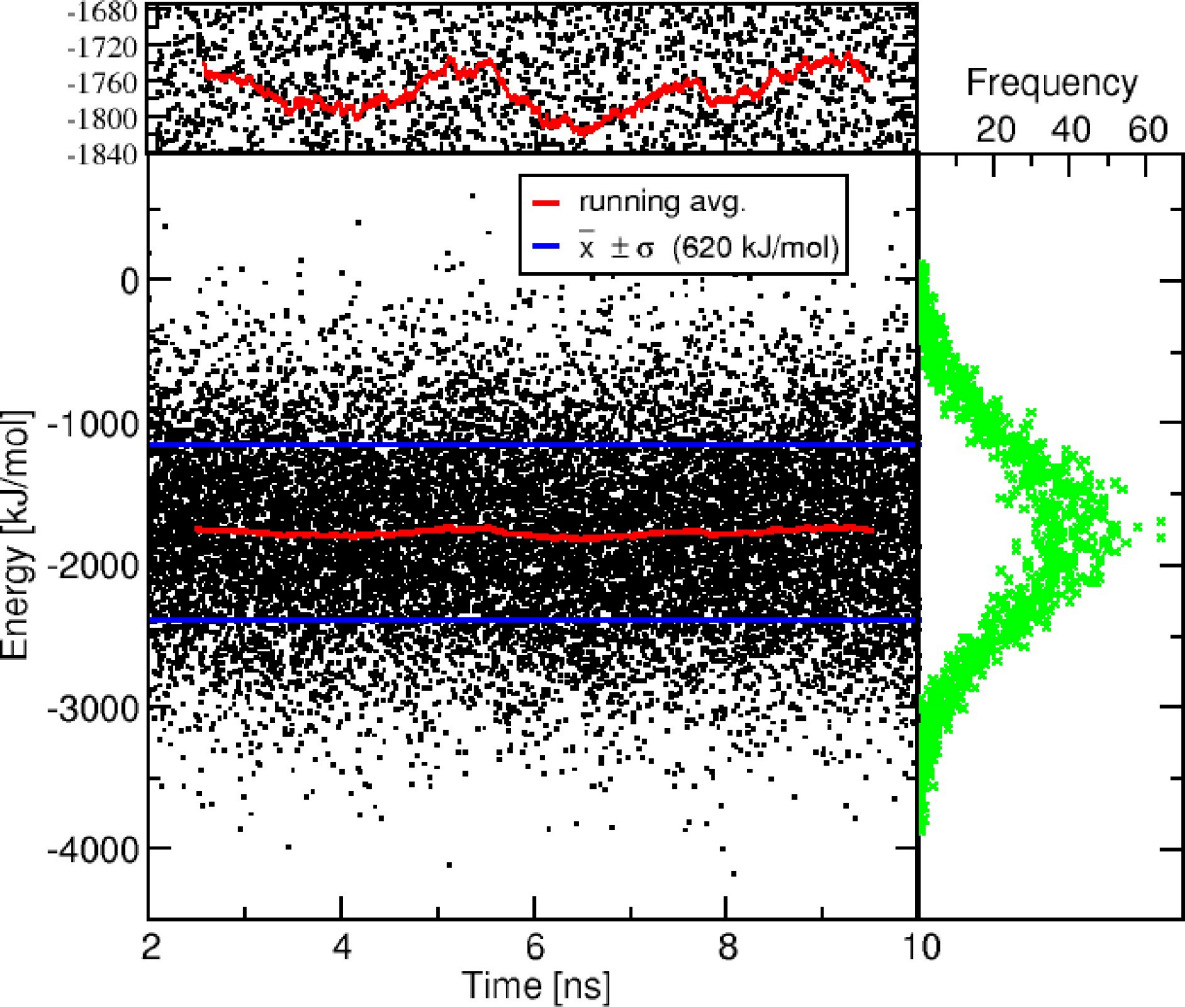
Energy fluctuation during the MD simulation was analyzed using histograms, running averages and the one‐sigma interval ($\bar{x}\pm \sigma$ ).

Figure 5 shows the time evolution of the total energy during a typical MD simulation. The energy fluctuates in a range of around 4000 kJ/mol, while the one‐sigma interval is about 1200 kJ/mol. Since our goal is to evaluate the conversion relationship between the P20 energies and the MD energies, we first want to investigate the accuracy of the determination of the average energy based on a 10 ns MD simulation. In other words, we want to check how effective is the averaging of the considerable instantaneous total energy fluctuations during the MD runs, compared to the energy variations between the different P20 structures. As a simple estimate of the numerical error due to the averaging of the discrete energy values, we calculated the energy averages for a randomly selected subset of the MD snapshots with about half of the data set size.

We also calculated for the same data set the standard error of the mean (SEM $\sigma_{\bar{x}}$ ), which is given by:

(6) $$\sigma_{\bar{x}}\approx \frac{\sigma_{x}}{\sqrt{N}}$$

Since our energy data points are highly correlated at short times, it is not appropriate to use the number of MD steps for *N*. Instead, we propose to use the number of typical hydrogen bond lifetimes (10 ps for relaxation of the hydrogen bond network of liquid water) for this quantity; for a simulation time of 10 ns, this results in *N*=10 ns/10 ps=1000. The use of the longer relaxation times corresponding to the peptide groups would lead to a “more‐than‐local equilibration”, however our idea behind this entire backmapping approach is to leave the overall structure (as delivered by the coarse‐grained model) unchanged as much as possible (i. e. doing only a local equilibration to avoid steric incompatibilities).

Using our standard deviation σ =620 kJ/mol and the resulting N=1000 gives us an estimated energy error:

(7) $$\sigma_{\bar{x}}\approx \frac{620\;\mathrm{kJ}/\mathrm{mol}}{\sqrt{1000}}\approx 19.6\;\mathrm{kJ}/\mathrm{mol}$$

Thus, the formal statistical uncertainty for the calculation of average all‐atom energy for a given P20 starting structure during the MD simulation is obtained as ±20 kJ/mol. For comparison, using instead a time interval of 1 ps for the assumed lifetime of a given MD simulation would give an estimated energy error of only 6.2 kJ/mol. It is interesting to note, that another estimate can be obtained visually from the running average (red line) in Figure 5. A closer inspection reveals fluctuations of about ±40 kJ/mol, which is in a similar range to the estimate from Eq. (7).

**Figure 6:**
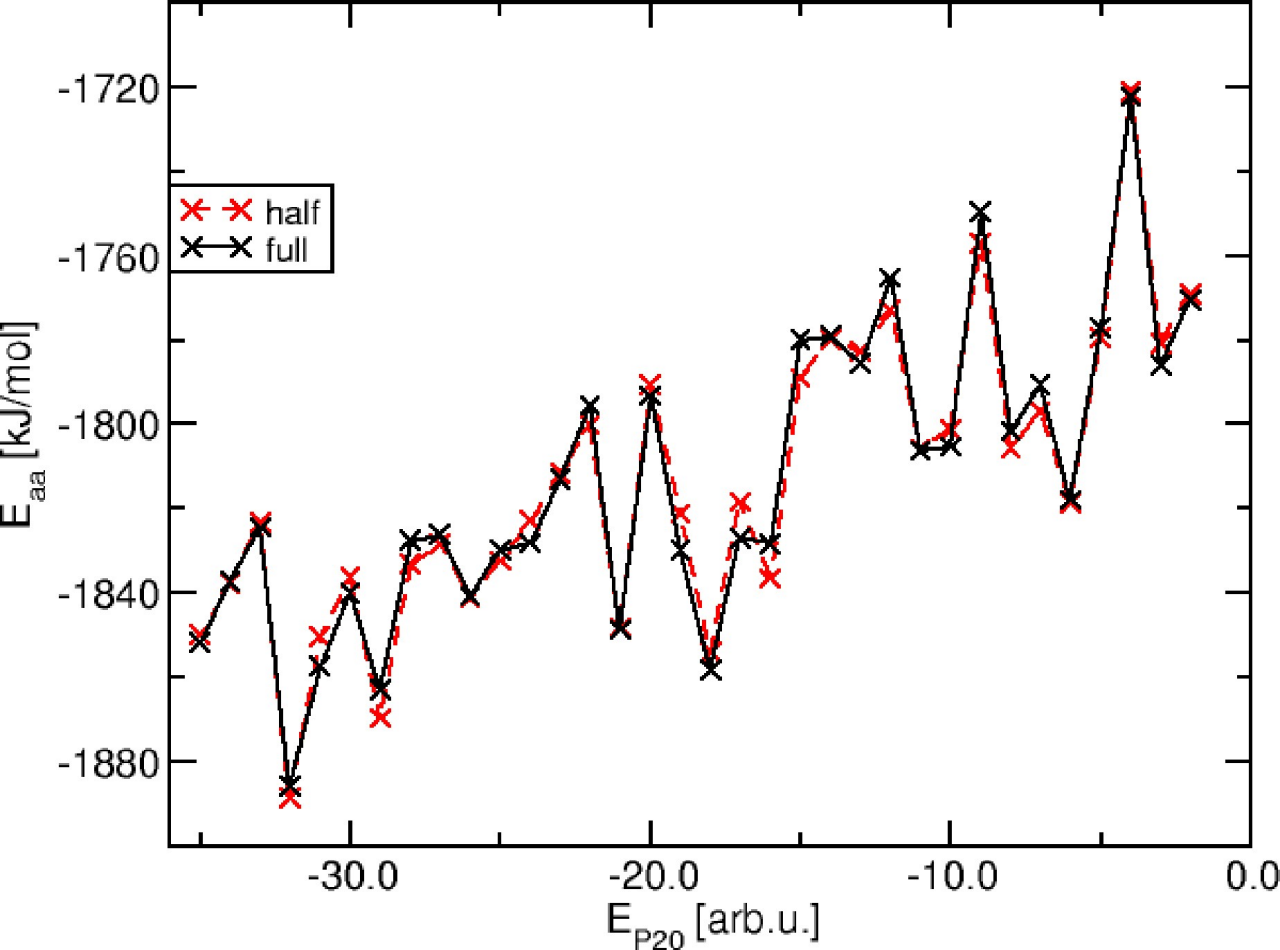
Validation of the statistical averaging accuracy from our MD simulations.

In Figure 6 the average atomistic total energy for a series of PRIME20 converted structures is shown for two averaging protocols: first using all MD snapshots (black) or only half of the available number of snapshots (red), selected randomly from the entire MD trajectory. This comparison is intended to illustrate the accuracy of the statistical averaging from a different perspective.

The averaging error this way turns out to be considerably smaller than the statistical error obtained previously (see Figure 5) derived from the explicit energy distribution. Therefore, we believe that our energy averaging protocol based on the 10 ns MD simulations is sufficient to yield converged average energy values with an accuracy around 5 kJ/mol. We want to stress, that this is not an accurate statistical error but rather a consistency check that not obvious bias is generated by our approach.

## Conclusions

We have determined the energy scale conversion factors from the coarse‐grained protein interaction model PRIME20 to all‐atom energies at the common force‐field level using explicit solvation and local conformational equilibration. Using a previously established protocol for the structure conversion,^[7]^ we have generated an ensemble of conformations using stochastic approximation Monte Carlo sampling. We subsequently computed atomistic energies for each value of the coarse‐grained interaction descriptor (peptide backbone hydrogen bonding and sidechain interaction) by averaging over a set of about ten different coarse‐grained conformations, equilibrating each conformation for about 10 ns via molecular dynamics simulations.

Our central result is that the atomistic physical energy scale for the backbone hydrogen bonding interaction of the PRIME20 model (which uses dimensionless energy units) is obtained as 2 kJ/mol per backbone interaction and virtually zero per sidechain interaction. This energy scale appears comparably small at first sight but is explained in terms of its interpretation as relative energies with respect to competing interactions (peptide to solvent). Our results confirm previous findings about salt bridges in peptides.^[38]^ We validate our findings by carefully estimating our statistical errors in the determination of the average atomistic energy values using several statistical techniques. Eventually, our results will allow for an insightful interpretation of structures generated using the coarse‐grained PRIME20 interaction model.

## Conflict of Interests

The authors declare no conflict of interest.

1

## Supporting information

As a service to our authors and readers, this journal provides supporting information supplied by the authors. Such materials are peer reviewed and may be re‐organized for online delivery, but are not copy‐edited or typeset. Technical support issues arising from supporting information (other than missing files) should be addressed to the authors.

Supporting Information

## Data Availability

The data that support the findings of this study are openly available in GitHub at https://github.com/thomascookies/Reverse‐mapping‐of‐coarse‐grained‐polyglutamine‐conformations‐from‐PRIME20‐sampling, reference number 1.
