# Supplementary material for: Assignment of a Physical Energy Scale for the Dimensionless Interaction Energies within the PRIME20 Peptide Model
Source: Chemphyschem. 2024 Oct 30;25(24):e202400592. doi: 10.1002/cphc.202400592 (PMC11648838; doi:10.1002/cphc.202400592)
Supplement: Supplementary file 1 — Supporting Information [file CPHC-25-e202400592-s001.pdf]

ChemPhysChem

Supporting Information

Assignment of a Physical Energy Scale for the Dimensionless Interaction Energies within the PRIME20 Peptide Model

Thomas Kunze, Christian Lauer, Christian Dreßler, and Daniel Sebastiani*

Supporting Information

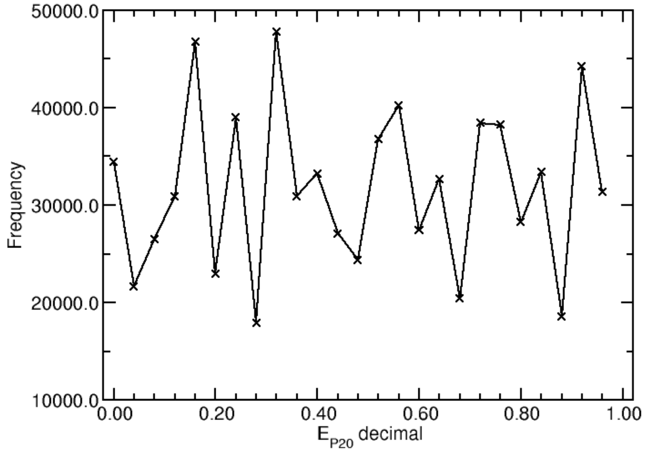

Figure S1: Distribution of the decimals of the E_{P20} of all coarse-grained structures.

In Fig. S1, the distribution of all coarse-grained structures is shown in order of decimal number, thus eliminating the possibility of over-representation of a few decimal energies.
In Fig. S2 the energy distribution is shown for 100 different structures with the same PRIME20 energy. Additionally, a Gaussian fit was applied to one bin setting.

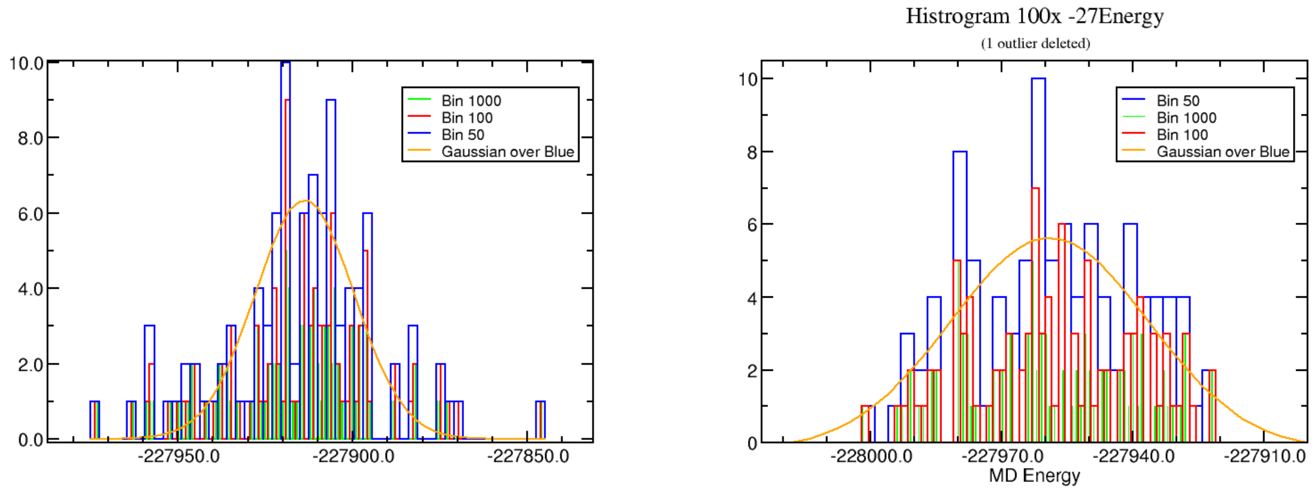

Figure S2: Histogram and Gaussian fit of the average MD energies for a series of converted coarse-grained structure with equal energies $E_{P20} = -8$ (left) and $E_{P20} = -27$ (right).

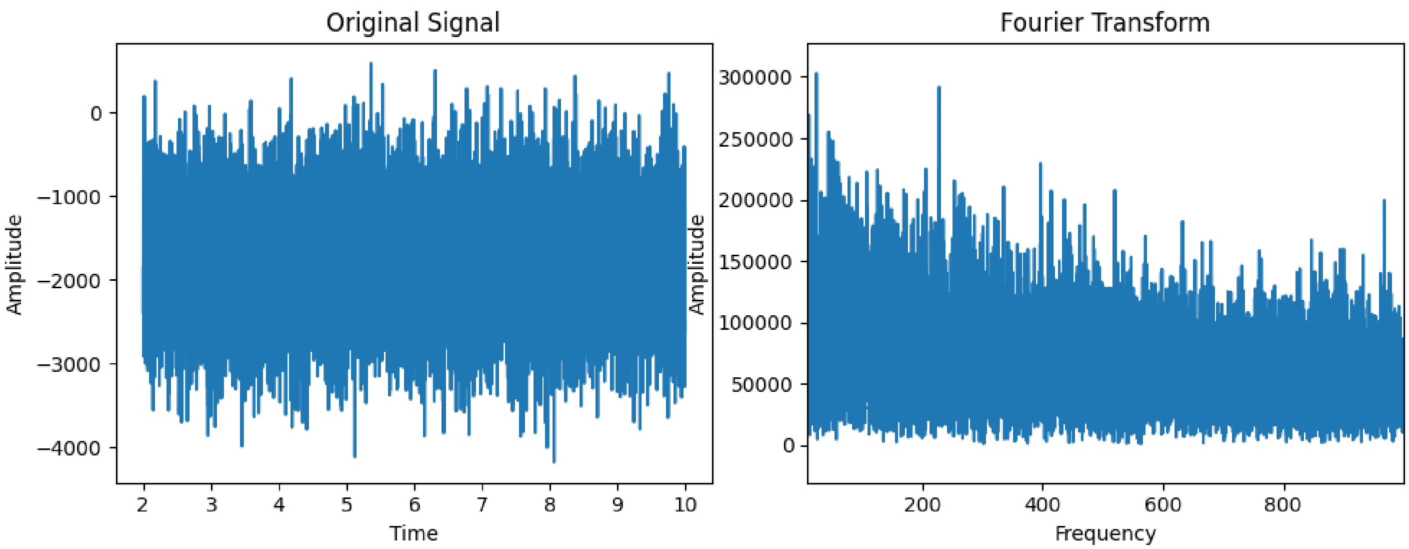

Figure S3: Fourier-Transformation of the temporal energy progression during a typical MD simulation.

Fig. S3 illustrates the energy progression during a typical MD simulation. The Fourier-transformed graph shows no distinct frequencies, indicating that the energy is not strongly dependent on frequency.

Githublink

https://github.com/thomascookies/Reverse-mapping-of-coarse-grained-polyglutamine-conformations-from-PRIME20-sampling

All structures are located in the subdirectory:
E_scale_assingment_structures
